# The Ultrasonography Characteristics of Borderline Ovarian Tumor Subtypes

**DOI:** 10.1002/jum.16756

**Published:** 2025-06-30

**Authors:** Roni Yoeli‐Bik, Ernst Lengyel, Ilan E. Timor‐Tritsch, Katherine Kurnit, Serghei Puiu, Ryan E. Longman, Jacques S. Abramowicz

**Affiliations:** ^1^ Department of Obstetrics and Gynecology The University of Chicago Chicago Illinois USA; ^2^ Department of Obstetrics and Gynecology Helen Schneider Hospital for Women, Beilinson Rabin Medical Center Petach Tikva Israel; ^3^ Department of Obstetrics and Gynecology Icahn School of Medicine at Mount Sinai New York New York USA; ^4^ Department of Radiology and Imaging “Nicolae Testemitanu” State University of Medicine and Pharmacy/Repromed Plus Hospital Chisinau Republic of Moldova

**Keywords:** borderline tumor, ovarian epithelial carcinoma, ovarian neoplasm, ultrasound

## Abstract

Borderline ovarian tumors, which are of epithelial origin, exhibit malignant histological features without stromal invasion. They differ from invasive ovarian carcinomas since often diagnosed in younger patients, at an early stage, and have a more favorable prognosis. This allows a more conservative surgical approach for some patients who desire fertility‐sparing surgeries. Grayscale, color and power Doppler ultrasonography are the initial imaging modalities for characterizing adnexal masses and evaluating their risk of malignancy. This review summarizes the main sonographic features of borderline ovarian tumors that are useful for pattern recognition.

**Abbreviations**
BOT: Borderline ovarian tumor
US: Ultrasonography
LGSC: Low‐grade serous ovarian carcinoma
HGSC: High‐grade serous ovarian carcinoma
CDI: Color Doppler imaging
PDI: Power Doppler imaging
SMVI: Superb microvascular imaging
MRI: Magnetic resonance imaging
CT: Computed tomography
PET‐CT: Positron emisson tomography
AI: Artificial intelligence

Approximately 15% of all epithelial ovarian tumors are classified as borderline.^1^ These tumors harbor malignant histological features but do not invade the stroma and, therefore, have a more clinically indolent course than invasive ovarian cancers.^2^ The incidence of borderline ovarian tumors varies between 1.8 and 4.8 out of 100,000 women per year.^3^ Borderline ovarian tumors (BOTs) are a heterogeneous group that can be further sub‐classified into several subtypes, with serous and mucinous being the most prevalent. Serous BOTs are more commonly diagnosed in North America, Europe, and the Middle East, whereas mucinous BOTs are more frequent in East Asia.^4^ Seromucinous, endometrioid, clear cell, and Brenner BOTs are rare, accounting for <5% of all BOT cases.^5^

BOTs are often diagnosed more than a decade earlier than invasive epithelial ovarian cancers,^1^ and a third of the patients are younger than 40 years.^6^ The symptoms associated with the tumors are vague and non‐specific, such as abdominal pain and distention or possible torsion.^3^ Almost a third of patients are asymptomatic at the time of initial diagnosis.^7^

Accurate and reliable preoperative diagnostic methods that can differentiate between benign, borderline, and malignant adnexal lesions are needed to help guide effective management. Patients with adnexal masses suspected to be malignant on imaging require prompt referral to gynecologic oncologists in centers of medical excellence for improved outcomes.^8^ In contrast, presumed benign lesions in asymptomatic patients can be conservatively managed.^8^ Developing and refining such noninvasive imaging methods is challenging due to the high prevalence of benign adnexal masses, the rarity of BOTs and invasive ovarian carcinomas and their non‐specific clinical presentation, and how often the imaging features of different tumor subtypes overlap. Ultrasonography (US) remains the most important and available imaging modality for the initial characterization and risk stratification of adnexal lesions.^9^ Despite studies that have aimed to delineate unique sonographic appearances of BOTs,^10^, ^11^, ^12^, ^13^, ^14^, ^15^ ultrasound‐based diagnoses are confirmed postoperatively in only 29–69% of cases.^3^ A meta‐analysis found a mean sensitivity of 66% and a mean specificity of 85% for diagnosing a BOT on ultrasound imaging,^7^ emphasizing the great challenge of correct diagnosis. Even in the hands of expert ultrasound examiners, the correct classification of BOTs by pattern recognition was reported to have variable efficacy. In one study, only 44% of BOTs were correctly identified, compared with benign lesions (76%) and malignant cases (83%).^16^

Continuous efforts to standardize sonographic assessments, evaluate new imaging modifications and modalities, study novel biomarkers, and discern subtle differences between tumor subtypes will improve preoperative diagnostic accuracy. Until accurate combined imaging techniques and biomarkers are developed and validated, sonographic pattern recognition by expert ultrasound examiners will continue to play a significant role in preoperative tumor assessments. This review sets out to summarize the main sonographic features of borderline ovarian tumors that are useful in clinical assessments by experts and discuss the pitfalls and challenges of their differential diagnosis.

## Serous Borderline Ovarian Tumors

Serous borderline ovarian tumors are one of the most common BOT subtypes.^17^ They are characterized by epithelial proliferation with hierarchical papillary branching. Serous BOTs contain various stromal core components and are often associated with extra‐ovarian peritoneal implants with no stromal invasion.^17^ Less than 10% of all serous BOTs harbor a micropapillary/cribriform histology architecture.^18^ This pattern is a known risk factor for recurrence^19^ and the development of extra‐ovarian invasive implants, which are now a defining feature of low‐grade serous carcinomas.^17^

**Figure 1:**
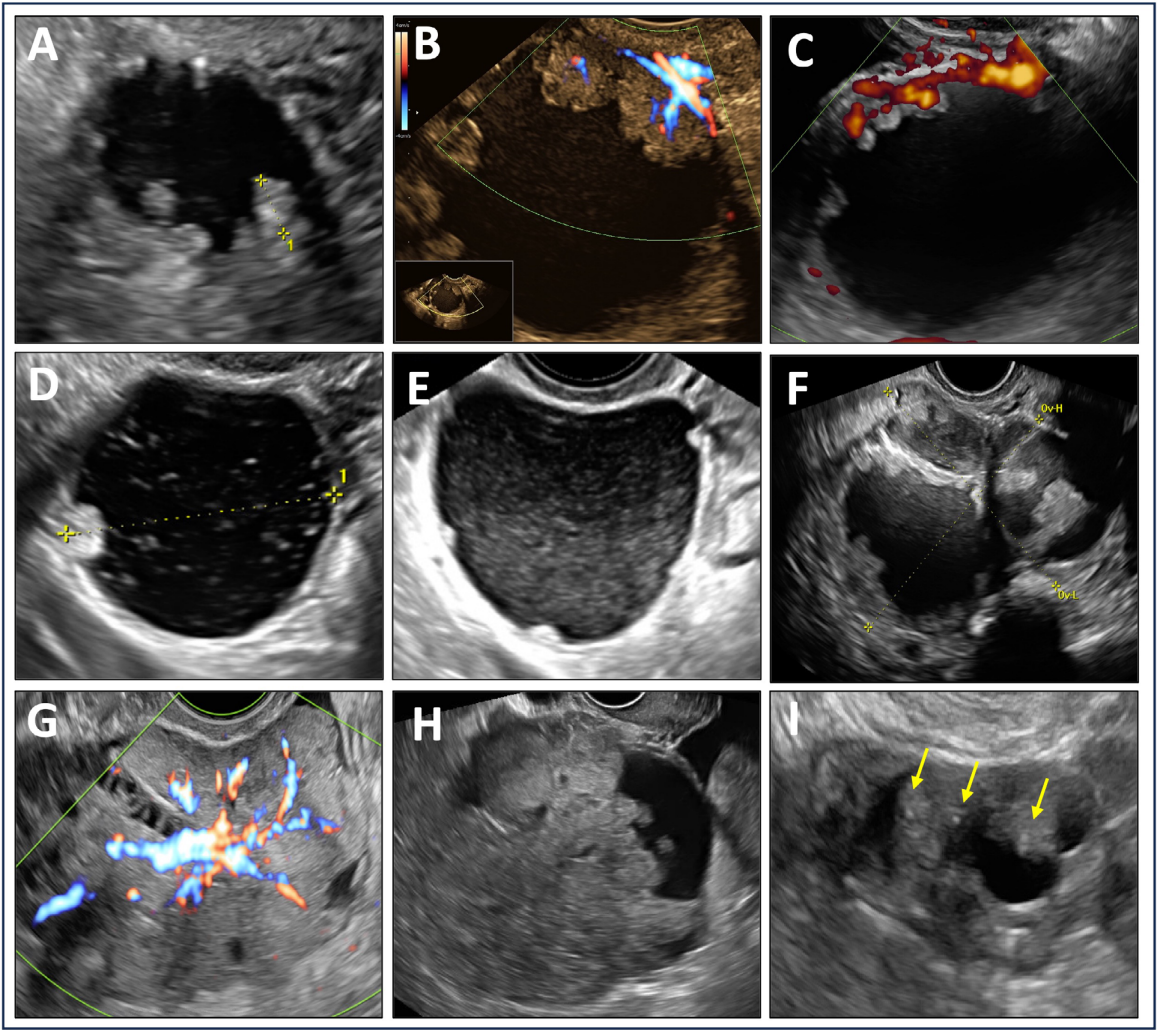
Serous ovarian tumors. Serous borderline ovarian tumors usually appear on ultrasonography Doppler imaging as cystic lesions with numerous vascularized papillary projections (**A–F**). Rarely, however, they may show an exophytic growth pattern with predominantly solid tumors that exhibit rich hierarchical vascular branching (i.e., a fireworks sign; **G**). Serous low‐grade ovarian cancer (**H, I**) often presents as irregular solid lesions with exophytic growth (**H**) or cystic lesions with endophytic papillary projections (**I**). Hyperechoic foci representing small calcifications may also be present (yellow arrows in **I**).

About one‐third of serous BOTs present bilaterally, and most appear on sonographic assessment as unilocular‐solid (or multilocular‐solid) lesions with numerous intra‐cystic irregular papillary excrescences^11^, ^12^, ^14^, ^20^ (Figure 1, A–F). These papillary projections are defined on ultrasonography as solid elements, at least 3 mm in height, that protrude into the cyst cavity and are surrounded by fluid.^21^ In serous BOTs, papillary projections do not exhibit acoustic shadowing and are often vascularized on Doppler imaging.^11^, ^15^, ^20^

The main differential diagnosis of serous BOTs includes benign cystadenofibromas and invasive epithelial ovarian cancers, both of which may also present as cystic lesions with papillary projections.^12^, ^15^, ^22^, ^23^ However, the typical solid papillary projections in benign cystadenofibromas are characterized by an avascular nature on Doppler imaging, and they often cast a posterior acoustic shadowing.^24^ In contrast, the papillary projections in serous BOTs and malignant ovarian lesions are not only often vascularized and have less prevalent acoustic shadowing, they also tend to have greater height and are more numerous, confluent, and disseminated than those papillary projections in benign tumors.^15^, ^22^ There are also some differential features on imaging that can help distinguish serous BOTs and invasive ovarian lesions. With increasing degrees of malignant invasiveness on the continuum from borderline to early‐ and late‐stage ovarian cancer, in invasive cancer, solid elements are a greater part of the overall tumor, solid components are more prominent, and ascites becomes more prevalent.^13^, ^15^ Nevertheless, it is often impossible to differentiate between BOTs and invasive ovarian carcinomas with ultrasound, especially in early‐stage cases. Differentiating between the two may be less important clinically than distinguishing between BOTs and benign ovarian lesions, since both BOTs and invasive ovarian carcinomas invariably require surgical intervention, even if they require a different surgical extent.

Rarely, serous BOTs show exophytic tumor growth patterns on the ovarian surface with papillary surface projections^25^ that are frequently associated with noninvasive peritoneal implants.^26^ On sonographic imaging, the exophytic pattern is described as a lobulated solid mass with a clear demarcation line between the tumor growth and the normal ovarian tissue.^25^, ^26^, ^27^ Microcysts and tiny calcifications may also be present.^26^ The solid tumors often show rich hierarchical vascular branching on Doppler imaging (i.e., Fireworks sign)^25^, ^26^ (Figure 1G). This exophytic growth pattern may also be associated with low‐grade serous ovarian cancers (LGSC), thus further complicating the ability to differentiate between serous BOTs and low‐grade ovarian carcinomas preoperatively.^28^

LGSC are primary invasive ovarian cancers of epithelial origin that account for <5% of all ovarian carcinomas.^29^ They often arise in association with serous BOTs and are characterized by slow progression and chemotherapy resistance.^30^ The chief difference between a serous BOT and LGSC is that LGSC harbors stromal invasion, resulting in reduced overall survival.^29^ On sonographic evaluations (Figure 1, H and I), LGSCs often present as bilateral multilocular‐solid or irregular solid lesions with exophytic tumor growth.^20^ Papillary projections with varied amounts of vascular flow on Doppler imaging may be present in about one‐third of patients,^20^ and hyperechoic foci representing small calcifications are common findings^17^, ^20^ (Figure 1I). Notably, it is almost impossible to differentiate LGSC from high‐grade serous ovarian cancer (HGSC) by ultrasound.

## Mucinous Borderline Ovarian Tumors

**Figure 2:**
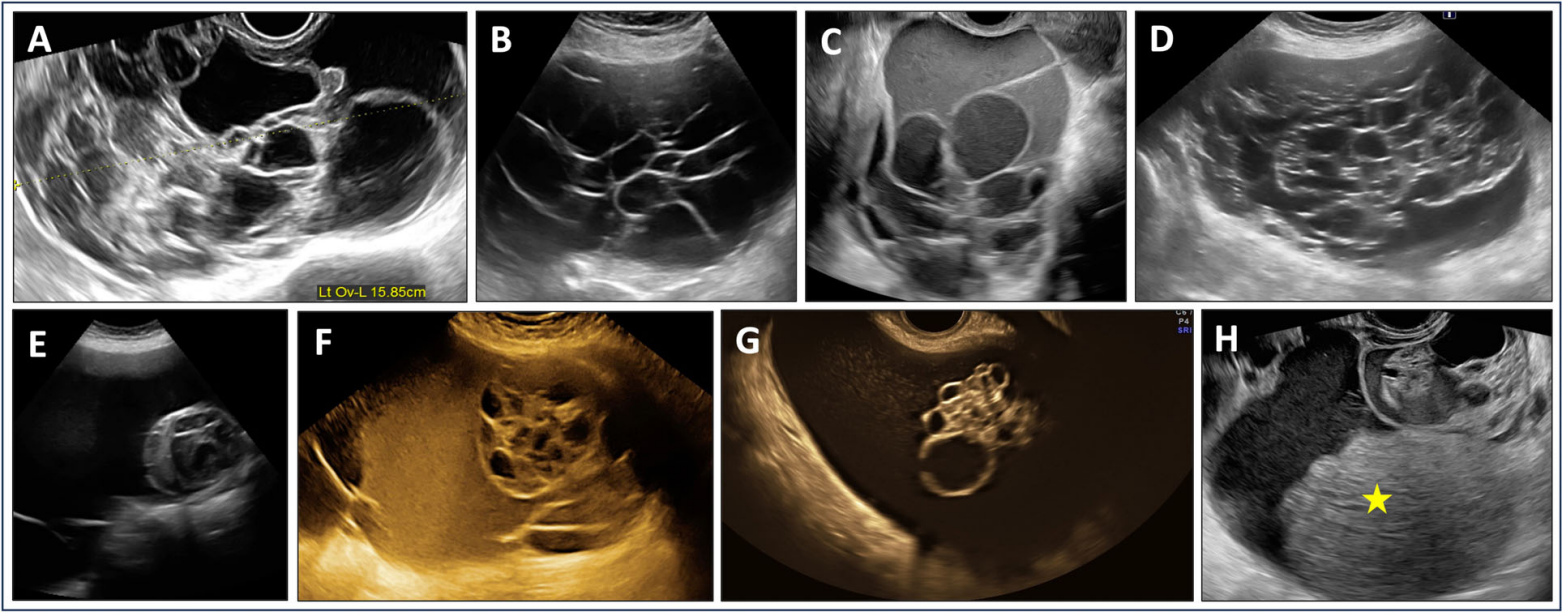
Mucinous borderline ovarian tumors. Mucinous borderline ovarian tumors are mostly unilateral, large multilocular cystic lesions containing more than 10 locules (**A–D**) with scattered low‐level echogenicity. Honeycomb nodules may be present (**E–H**). Rarely, a solid component may be visualized (yellow star in **H**).

Mucinous borderline ovarian tumors (gastrointestinal differentiation type) are the most common BOT subtype in Asia, accounting for about 70% of all BOT cases, and the second most common subtype in North America and Europe.^17^ The mean age at presentation is 45 years^17^ and tobacco smoking is a known risk factor.^4^ Mucinous BOTs may arise from mucinous cystadenoma in a stepwise progression and may present in association with benign ovarian teratomas or Brenner tumors.^17^ On sonographic examinations (Figure 2), mucinous BOTs are unilateral, very large (median diameter 20 cm) multilocular lesions. They sometimes contain more than 10 cyst locules^11^, ^31^ (Figure 2, A–D), with scattered low‐level echogenicity correlating with the thick gelatinous material seen on macroscopic examinations.^14^, ^31^ A honeycomb nodule (Figure 2, E–H), defined as a multilocular nodule arising from the inner cyst wall, is a characteristic sonographic finding but is not always present.^14^ Rarely, mucinous BOTs contain solid components that cause them to resemble mucinous carcinomas^14^, ^31^ (Figure 2H).

The mucinous tumor subtypes can pose diagnostic and therapeutic challenges due to their rarity, large size, and varied degree of differentiation.^32^ The sonographic appearance of mucinous BOT overlaps with that of invasive mucinous ovarian carcinomas, which often present as large lesions with more than 10 cyst locules and prominent vascularized solid components.^31^ In comparison, benign mucinous cystadenomas mostly appear as unilateral large multilocular lesions that typically harbor less than 10 cyst locules.^31^ Additional differential diagnoses include secondary metastases to the ovaries from primary colorectal, appendix, or biliary tract cancers since these are predominantly cystic lesions with numerous cyst locules and solid components (as compared with metastases arising from primary breast, uterine, or gastric cancers and lymphomas that are usually solid lesions).^33^ However, secondary metastatic tumors are often smaller (<10 cm) and tend to have bilateral involvement.^32^

**Figure 3:**
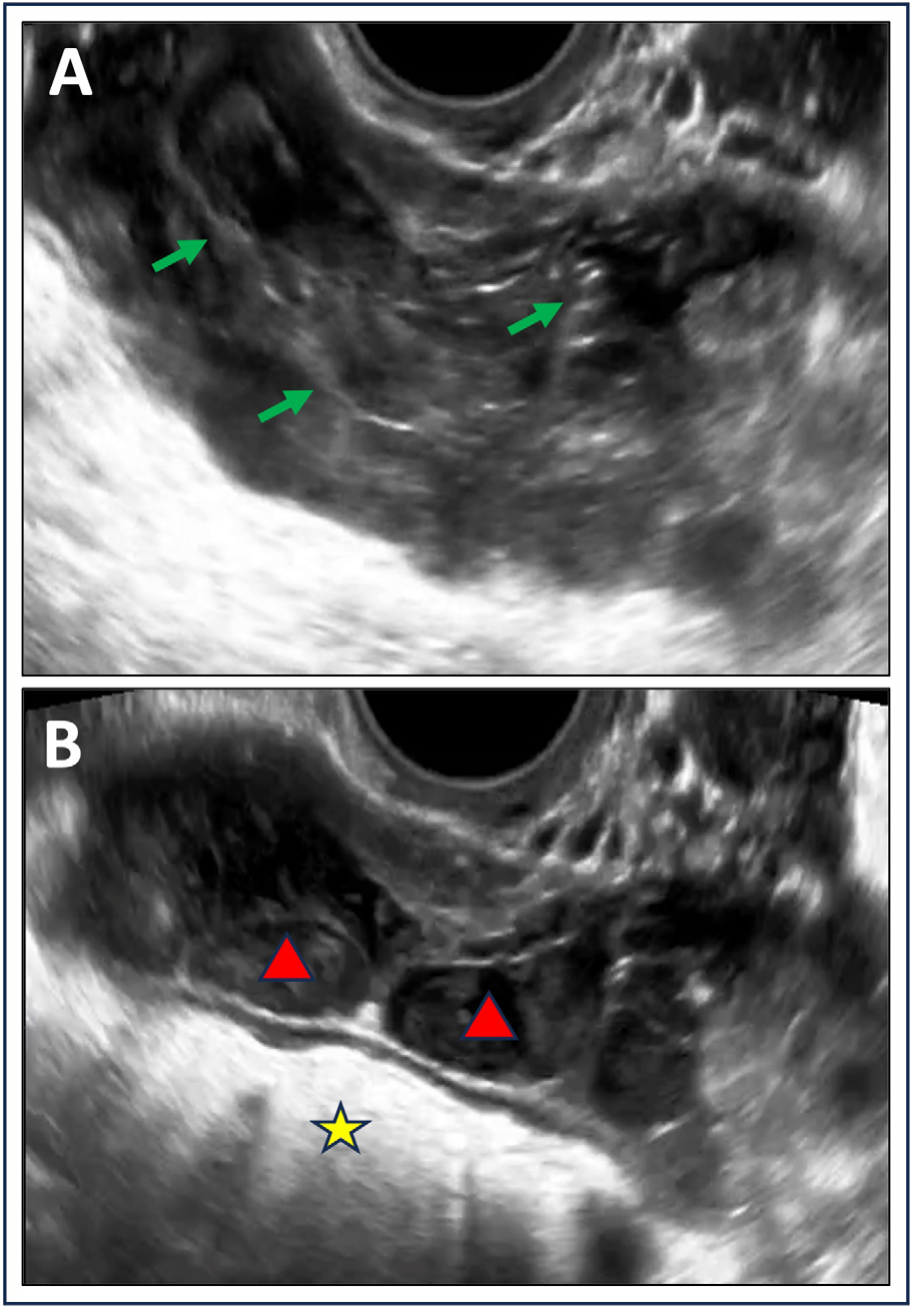
Pseudomyxoma peritonei. Pseudomyxoma peritonei often appears as echogenic ascites with diffused septations (green arrows in **A** and red triangles in **B**) and centrally displaced fixed bowels (yellow star in **B**).

A rare condition, pseudomyxoma peritonei (PMP), is usually associated with appendiceal mucinous neoplasia.^34^ A few reports indicate that it can also be rarely associated with mucinous BOTs arising in ovarian teratomas.^35^ Therefore, a thorough histopathologic examination of the appendix is almost always necessary for PMP cases due to the possibility of a primary appendiceal origin with secondary ovarian involvement. PMP is characterized by extensive dissemination of mucin content in the peritoneal cavity that may lead to bowel obstruction. On sonographic evaluations (Figure 3), it often appears as echogenic ascites with centrally displaced fixed bowels (i.e., starburst sign)^36^ caused by the involvement of the small bowel mesentery with the tumor. On ultrasound, additional signs of pseudomyxoma peritonei include diffused septations, scalloping of the liver, and thickened irregular peritoneum with heterogeneous echogenicity with some small anechoic areas.^36^, ^37^

## Seromucinous Borderline Ovarian Tumors

**Figure 4:**
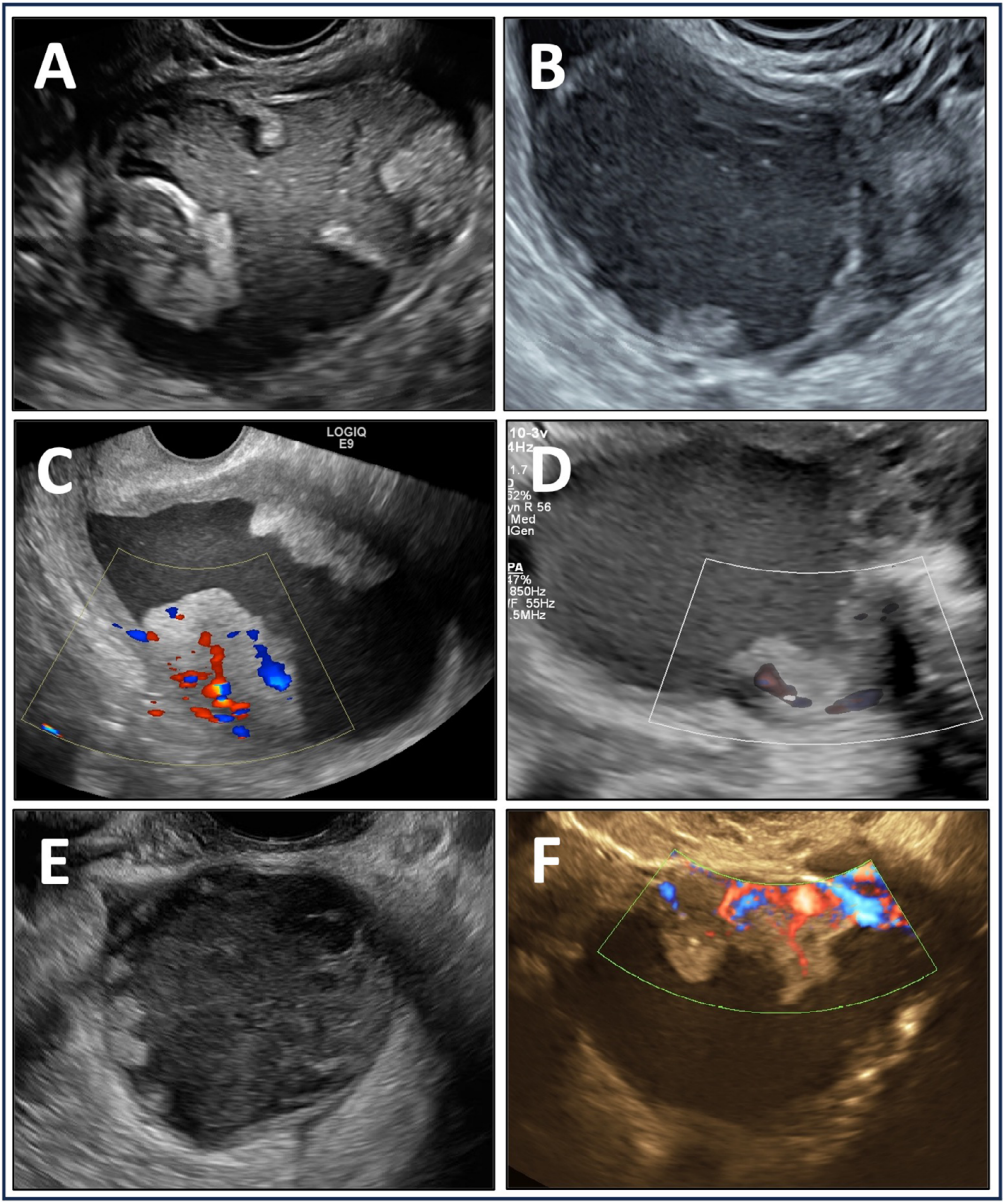
Seromucinous borderline ovarian tumors and differential diagnosis. Seromucinous borderline ovarian tumors are often cystic lesions with numerous vascularized papillary projections and ground‐glass cyst echogenicity, reflecting their association with endometriosis (**A–D**). Notably, in the differential diagnosis, atypical endometrioma may present as a cystic lesion with ground‐glass cyst echogenicity and papillary projections; however, it usually does not show vascular flow on Doppler imaging (**E**). In contrast, decidualized endometriomas during pregnancy may also contain papillary projections that are almost always highly vascularized on Doppler imaging (**F**).

Seromucinous borderline ovarian tumors, previously classified as mucinous endocervical‐type or Müllerian‐type, are defined as separate entities by the WHO ovarian tumors classification.^17^ Seromucinous BOTs may present with bilateral involvement and peritoneal implants and are often associated with endometriotic lesions.^38^ Their sonographic appearance generally resembles the serous BOT subtype (although on histopathologic examination, they differ)^39^ and primarily includes cystic lesions with numerous vascularized papillary projections.^3^, ^11^, ^31^ Seromucinous BOTs, however, often present with low‐level or ground‐glass cyst echogenicity^31^ (Figure 4, A–D), reflecting their association with endometriosis and, therefore, may also be confused with atypical endometriomas.^40^ A key difference is that the solid‐appearing elements in atypical endometriomas (Figure 4E) usually do not show vascular flow on Doppler imaging.^40^ Still, in many cases, it is impossible to distinguish atypical endometriomas from seromucinous BOTs without surgical evaluation. In addition, decidualized endometriomas (Figure 4F) during pregnancy may also contain papillary projections that, although often broad‐based and rounded with smooth surfaces, are almost always highly vascularized on Doppler imaging.^41^ Consequently, they may pose diagnostic and therapeutic challenges, especially when no prior scan documenting a typical endometrioma is available.^42^

## Rare Borderline Ovarian Tumors

Because endometrioid,^43^ clear cell,^44^, ^45^ and Brenner^46^, ^47^ BOTs are rare subtypes, the literature on their distinct clinical characteristics, typical imaging appearances, and outcomes is sparse. Unlike most other BOT subtypes, clear cell and Brenner BOT are usually diagnosed in postmenopausal women, and recurrence events are rare.^45^, ^47^

**Figure 5:**
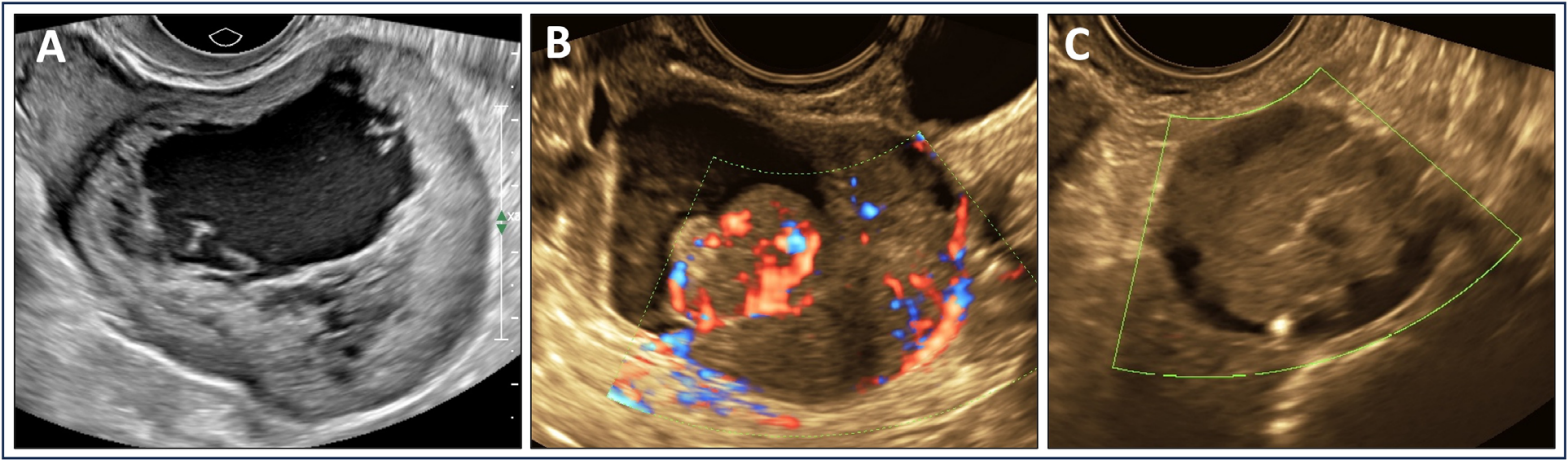
Rare borderline ovarian tumors. Clear cell and endometrioid BOTs are predominantly large solid tumors with small to large cystic areas on macroscopic examinations. Indeed, both the clear cell (**A**) and endometrioid (**B**) BOT cases showed large solid components with cystic areas and high vascular flow on Doppler imaging. Brenner BOTs macroscopic appearance is often described as cystic lesions with papillary projections. In this case (**C**), a cystic lesion with what appears to be a large papillary projection is present.

Endometrioid and clear cell BOTs are associated with endometriosis,^17^ and also frequently co‐occur with endometrial disorders such as endometrial hyperplasia.^43^, ^45^ Both endometrioid and clear cell BOTs are predominantly adenofibromatous in nature and on macroscopic examination are characterized as large solid tumors that might present with small to large cystic areas^17^, ^44^, ^48^, ^49^ (Figure 5, A and B). Rarely, endometrioid BOTs may exhibit intracystic architectural growth patterns presenting on sonographic imaging as cystic lesions with vascularized solid protruding components and ground‐glass cyst echogenicity,^17^, ^48^, ^50^ which may be thought to resemble the appearance of the seromucinous BOT subtype.

The sonographic appearance of Brenner BOTs is not well established (Figure 5C), because they are so rare. Their macroscopic appearance is often described as unilateral large cystic lesions with papillary projections, frequently accompanied by an adjacent solid fibrous component that represents synchronous benign Brenner tumors.^17^, ^46^, ^48^, ^49^ Patients with Brenner BOTs may present with synchronous urothelial tumors,^47^ which often appear on ultrasonography and Doppler imaging as vascularized intraluminal nonmobile irregular masses or as focal bladder wall thickening.^51^, ^52^ However, depending on the location and size, some bladder tumors are difficult to detect on ultrasonography.^51^

## Novel Borderline Ovarian Tumor Sonographic Marker

**Figure 6:**
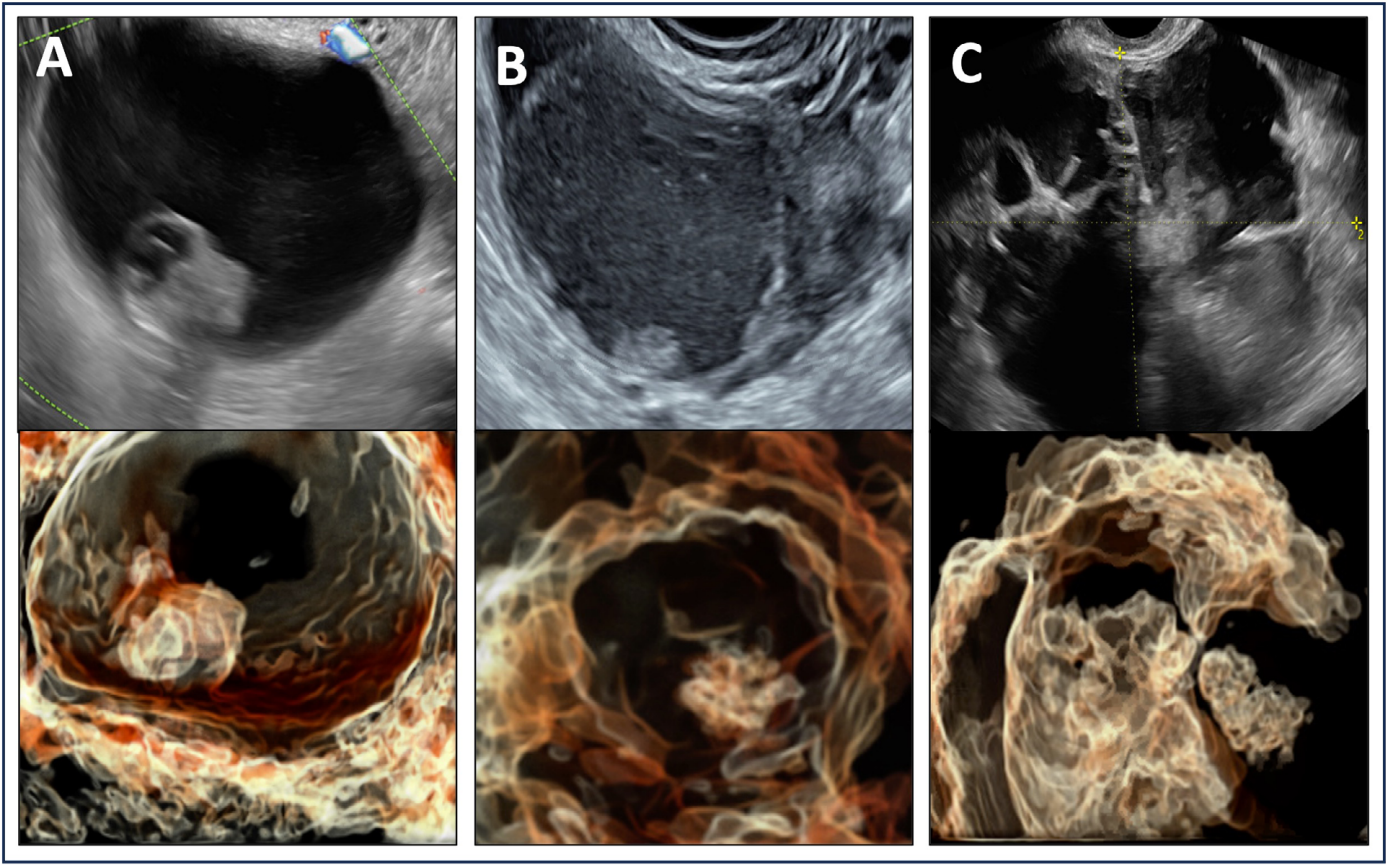
Borderline ovarian tumors and microcystic pattern. Ultrasonography microcystic pattern on grayscale (top row) and 3D‐silhouette mode (bottom row) in serous borderline (**A**), seromucinous borderline (**B**), and mucinous borderline ovarian tumors (**C**).

**Figure 7:**
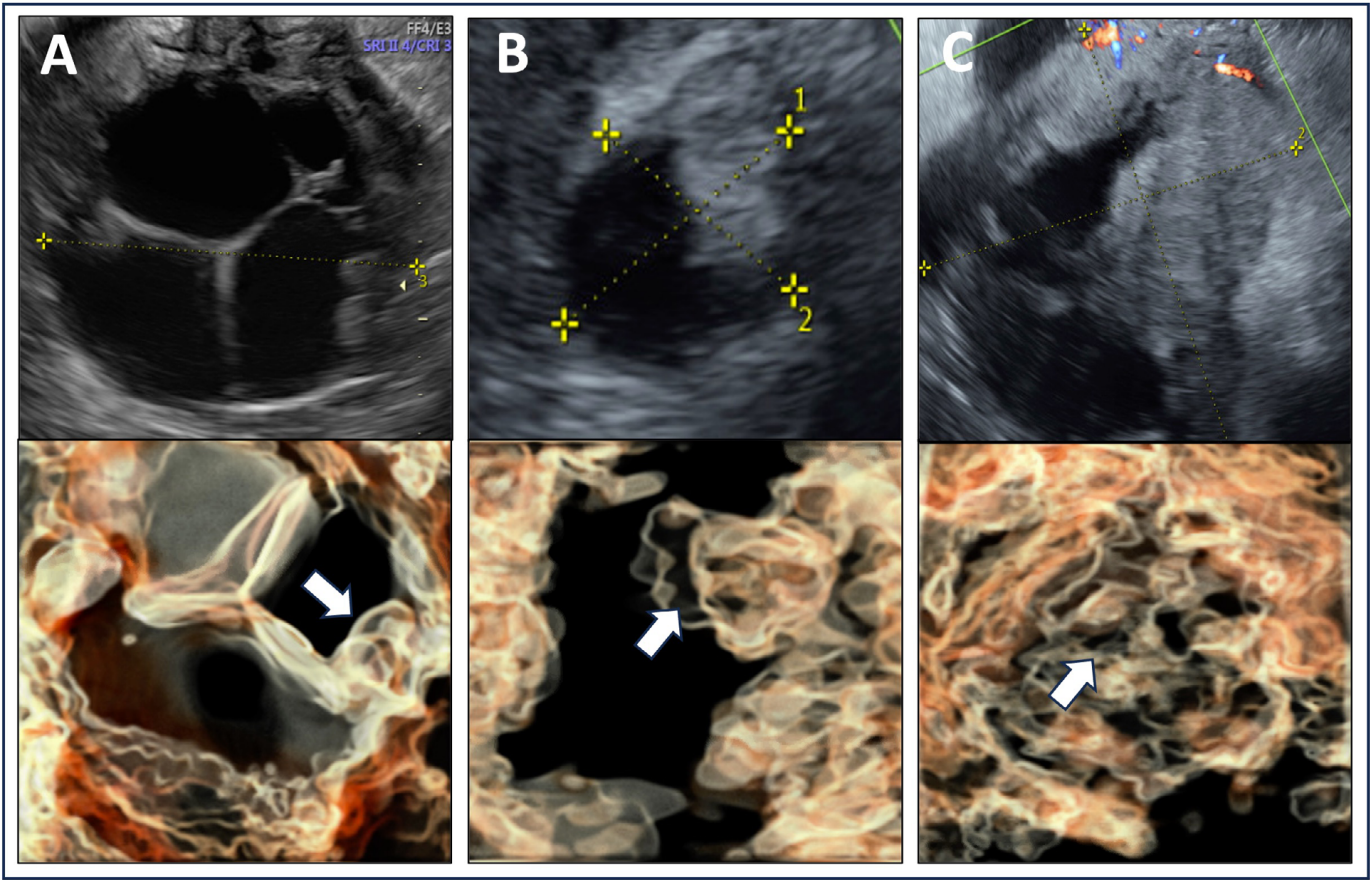
Differential diagnosis of borderline tumors with microcystic pattern. Ultrasonography grayscale (top row) and 3D‐silhouette mode (bottom row) of ovarian tumors presenting the microcystic pattern (white arrows) in benign cystadenofibroma (**A**), benign struma ovarii (**B**), and malignant invasive endometrioid carcinoma (**C**).

An accurate noninvasive diagnosis of BOT is very challenging since no tumor markers or distinct morphologic features can distinguish between borderline, benign, or malignant ovarian tumors with high sensitivity and specificity. Timor‐Tritsch and colleagues suggested that a microcystic pattern may be a novel sonographic marker of BOT cases.^53^ The microcystic pattern correlates with histopathologic evaluations and is defined as tiny 1 to 3 mm fluid‐filled, thin‐walled clusters of cysts found at papillary projections, solid elements, or tumor septation.^53^ A likely pathological explanation for a microcystic pattern is that it reflects multilevel papillary branching (which results in tissue gaps) and edematous areas within the stroma.^23^, ^54^, ^55^ Further sonographic evaluation of the microcystic pattern may be achieved using the ultrasonography 3D silhouette rendering mode^56^ (Figure 6). This technique utilizes the changes in the acoustic impedance of the tissues to construct a simultaneous display of the inner core and structures and the outer and back walls in a “see‐through” fashion. In recent studies, the microcystic pattern has been validated as an independent predictor of BOTs.^54^, ^55^ However, it has also been observed, albeit infrequently, in malignant lesions (most often invasive epithelial tumors) and in benign lesions (most often cystadenofibromas)^23^, ^54^, ^55^ (Figure 7).

## The Use of Superb Microvascular Color Doppler in Sonographic Evaluation of Borderline Ovarian Tumors

Conventional Doppler imaging techniques are standard for detecting vascularity typical of malignant tumors. In color Doppler imaging (CDI), a filter is used to prevent random motion and noise artifacts. The downside of CDI is that low‐amplitude flow cannot be detected. In conventional CDI, motion represses the low blood flow signals, and the image only includes blood flow with high amplitudes; therefore, in some cases, CDI is insufficient for accurate estimation of flow parameters and tumor diagnosis. In contrast, power Doppler imaging (PDI) can surpass these random artifacts, improving sensitivity to signals from small blood vessels with lower velocities.^57^ Still, some small velocities are hard to depict using traditional PDI.

**Figure 8:**
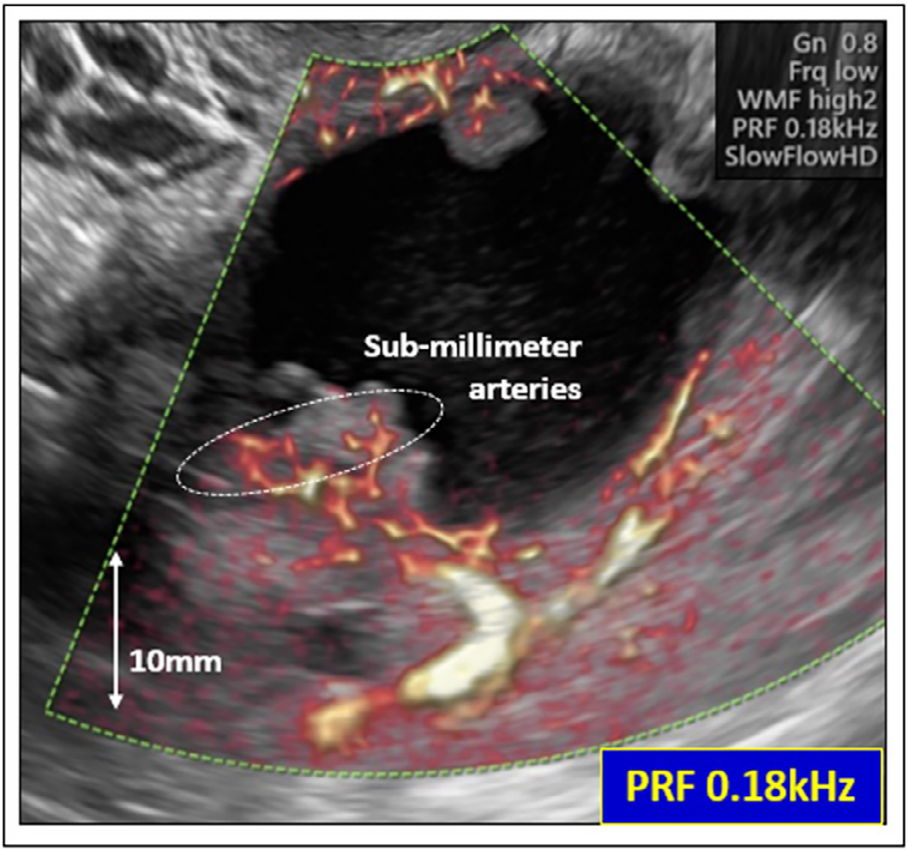
Example of microvascular imaging presenting extremely small, thin blood vessels of the size of arterioles in a borderline ovarian tumor using a 9–5 MHz transvaginal ultrasound transducer set to a pulse repetition frequency of 0.18 kHz.

Superb microvascular imaging (SMVI), also known as microvascular imaging or microvascular flow imaging, is a novel, noninvasive PDI technique that presents contrast‐free imaging of small vessel blood flow. This imaging modality applies advanced tissue clutter‐filtering capable of separating slow blood flow from artifacts, improving the sensitivity of visualizing flow at the level of thin vessels^58^ (Figure 8). The technology is readily accessible on most new US machines, making it easy to trial in different clinical scenarios.^59^, ^60^ For this reason, it has been suggested to be invaluable in the detection, diagnosis, and monitoring of disease.

**Figure 9:**
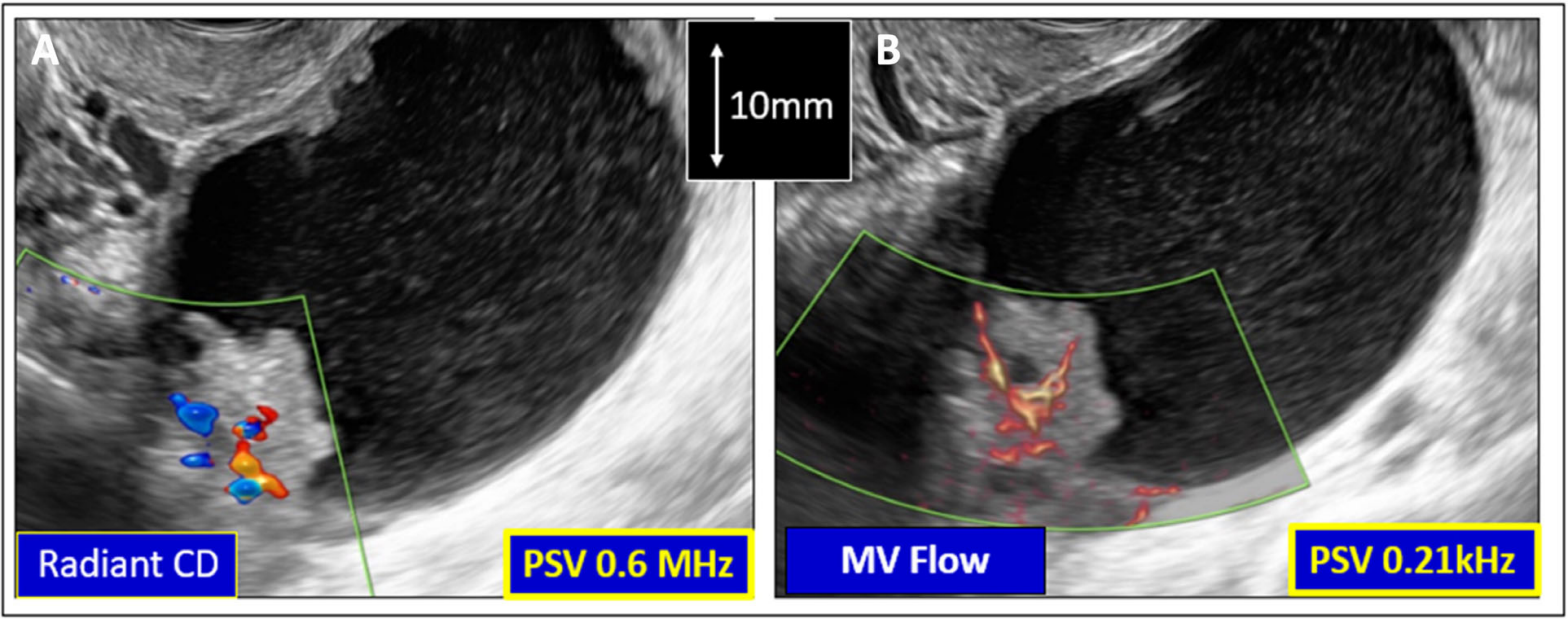
Ultrasound imaging of serous borderline ovarian tumor using radiant color Doppler US (CDUS) with a PRF of 0.6 kHz and microvascular imaging (MVI) using a PRF of 0.21 kHz. Note the difference in the details of the vascular patterns.

**Figure 10:**
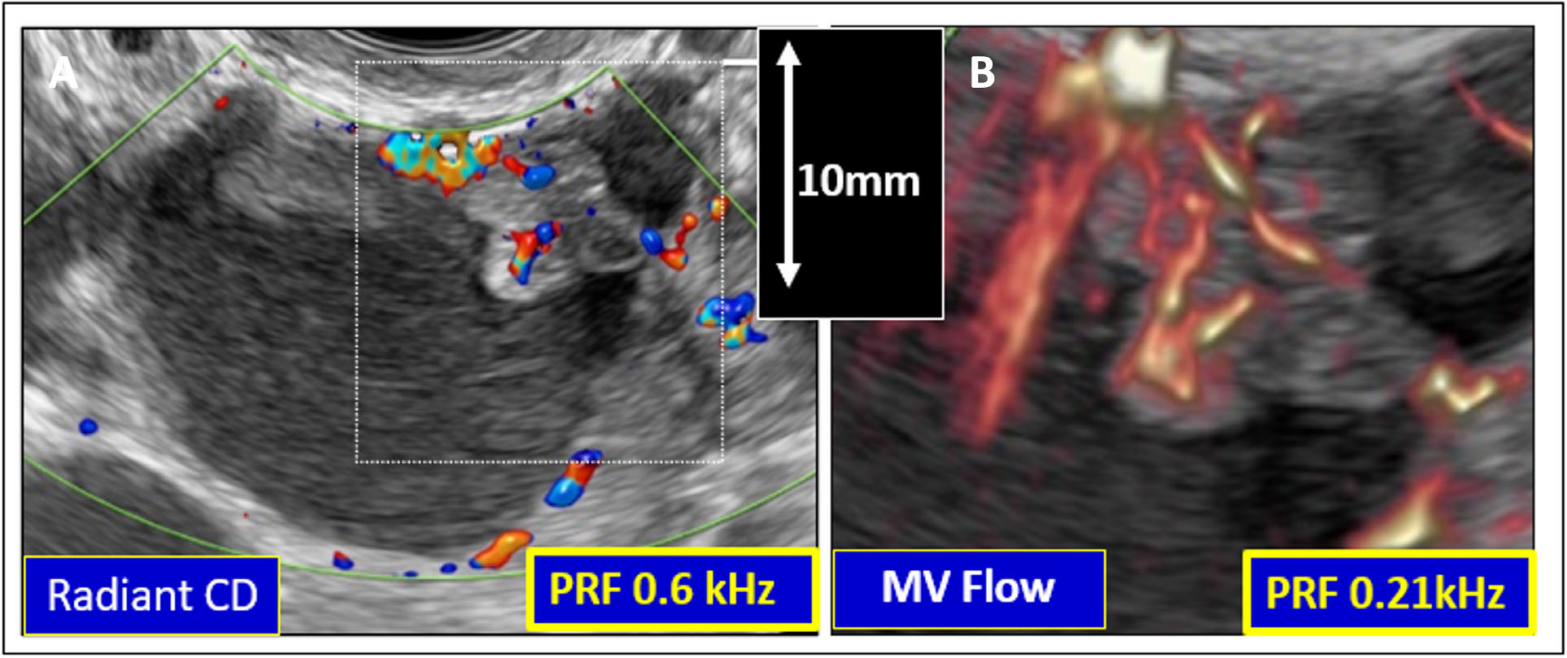
Ultrasound imaging of mucinous borderline ovarian tumor by radiant color Doppler US (CDUS) using a PRF of 0.6 kHz and microvascular imaging (MVI) using a PRF of 0.21 kHz. Note the difference in the details of the vascular patterns.

Superb microvascular imaging (SMVI) has also been introduced in obstetrical and gynecological imaging.^61^ Combined with a high‐frequency (6–12 MHz) transvaginal ultrasound probe, SMVI may provide an additional effective imaging technique for diagnosing primary or recurrent ovarian tumors and, more specifically, BOT (Figures 9 and 10). More importantly, when periodic follow‐up is instituted for women with previously treated BOT, the earliest detection of a sub‐centimeter recurrence in an otherwise normal‐sized ovary is crucial and might be possible using the SMVI technique. Although it has been found to be effective in research settings, its role in routine clinical practice is yet to be established. Large multicenter studies should be conducted to estimate SMVI's effectiveness in assessing tumor vascularity patterns and its diagnostic accuracy for BOT compared with conventional imaging techniques to potentially improve preoperative diagnosis.

## The Use of Magnetic Resonance and Computed Tomography Imaging Following Sonographic Evaluations of Borderline Ovarian Tumors

Ultrasound should be the first‐line imaging modality for adnexal lesions, but magnetic resonance imaging (MRI) can aid in their further characterization following indeterminate results on ultrasonography.^62^, ^63^ MRI's superior soft tissue resolution can increase diagnostic specificity and decrease false‐positive findings.^49^, ^64^ Several protocols have been suggested to improve lesion diagnosis using more consistent approaches and morphological‐functional imaging techniques.^48^, ^64^, ^65^, ^66^ Characteristic morphological patterns reported to correlate with BOTs include hierarchical papillary branching (i.e., sea anemone‐like pattern) at T2‐weighted MRI in serous and seromucinous BOTs; and hypointense microcysts on T2‐weighted MRI with reticular enhancement on contrast‐enhanced T1‐weighted MRI in mucinous BOTs.^48^, ^67^ Additional MRI features that may aid in distinguishing mucinous BOTs and carcinomas from benign mucinous cystadenoma include fluid signal intensity that is high on T1W and low on T2W MR imaging.^48^ However, all these features may also be present in some benign and early‐stage invasive epithelial cancers.^48^ Integration of the MRI diffusion‐weighted and apparent diffusion coefficient techniques might improve BOT diagnosis,^48^, ^64^, ^68^ but their usefulness in clinical decision‐making is yet to be established.^9^ Computed tomography (CT) imaging is widely available and frequently used for preoperative surgical planning to gauge the spread of disease, especially in the upper abdomen. Still, its role in differentiating a benign from a malignant or borderline mass is limited.^49^ While positron emission tomography CT (PET‐CT) has high sensitivity in characterizing lymph node metastases, it is not used for the characterization of adnexal tumors due to its limited resolution.^9^

## Discussion and Future Directions

Most (75%) borderline ovarian tumors are detected at an early stage when they are confined to the ovary, resulting in a favorable prognosis with an overall 10‐year survival rate exceeding 95%.^1^, ^5^ Therefore, women diagnosed in their reproductive years with early‐stage tumors might be good candidates for fertility‐sparing surgeries, which allow the preservation of one or both ovaries and the uterus.^69^ The risks, relapse rates, and personal fertility desire must be carefully considered and discussed between the patient and her gynecologic oncologist. The recurrence rate after fertility‐preserving surgeries for BOT (which varies between 5 and 34%) is higher than the recurrence rate after more radical surgeries (reported to be between 3.2 and 7%).^69^ However, with close surveillance and extended follow‐up, most recurrent BOT cases after ovarian preserving approaches are safely managed surgically with good oncologic outcomes.^70^ Recurrence rates are also reported to be higher for patients with advanced FIGO stages at diagnosis and in the setting of residual disease after surgery.^2^, ^71^, ^72^

Approximately 4 to 7% of women with a serous BOT will develop invasive cancer, most commonly a low‐grade serous carcinoma, sometimes through a micropapillary intermediary step in the epithelial compartment.^5^, ^73^, ^74^ Similarly, invasive mucinous ovarian carcinomas, another subtype of epithelial ovarian cancer, may derive from mucinous BOTs.^5^ Shared molecular and genetic characteristics of borderline and subsequent invasive epithelial ovarian tumors suggest a continuum of disease in a pathway of stepwise progression.^30^

BOTs have a high survival rate and rarely require radical surgery; thus, reliable preoperative diagnosis is of vital clinical importance. Current imaging tools are insufficient for accurate diagnosis. Several imaging modifications, such as contrast‐enhanced ultrasonography,^64^, ^75^, ^76^ photoacoustic imaging,^77^, ^78^, ^79^ and elastography,^80^, ^81^ have been explored, but none have yet been found to be significantly effective, especially for BOT diagnosis. Recent work on artificial intelligence (AI) based tools for ovarian cancer diagnosis has shown promising initial results.^82^, ^83^, ^84^ Novel biomarkers are being examined to improve accurate diagnosis. Eventually, a combined imaging and biomarkers approach will hopefully transform how patients with adnexal masses, and specifically BOTs, are managed to improve their care.

Given the low prevalence of borderline and invasive ovarian cancers, collaborative efforts are needed to establish integrative tailored models for more consistent adnexal mass evaluations, which will result in improved patient outcomes. We should aim for standardized sonographic evaluations, counsel with expert ultrasound examiners for indeterminate cases, and utilize complementary imaging techniques such as MRI in patients with inconclusive results. Familiarizing oneself with the subtle differences in sonographic findings between BOT subtypes captured by subjective evaluations can aid in better counseling patients and their gynecologic oncologists in the decision‐making process.

## Data Availability

Data sharing is not applicable to this article as no new data were created or analyzed in this study.
